# Medical Student Perspective on Palliative Care Teaching

**DOI:** 10.1177/1049909120922971

**Published:** 2020-05-01

**Authors:** Amandeep Pahal, Mohini Bhagalia, Shivani Desai, Bianca Goonewardena

**Affiliations:** 1 Medical School, St George’s University of London, United Kingdom; 2 Medical School, University of Leicester, United Kingdom; * All authors have equal contribution

As current medical students, we were deeply interested in the recent article on the implementation of a palliative care elective for fourth-year medical students.^1^ Intriguingly, there are differences in palliative care between medical schools. We took this opportunity to reflect upon the end-of-life teaching in our curricula.

The article demonstrates implementing a palliative care elective proved to be beneficial for medical students.^1^ However, despite this, it is important that medical students optimize the opportunities provided during medical school. Throughout medical school, we have had ample opportunities to observe clinicians engaging in a variety of difficult conversations. Examples of such situations include do-not-attempt cardiopulmonary resuscitation orders and advance care planning. Speaking to patients, families, and members of staff has made us more confident when discussing the decisions that must be taken as well as coping with death in the workplace.

We have experienced many forms of teaching in this field at our respective universities. These include workshops for breaking bad news, simulation scenarios with clinicians, and hospice rotations. These experiences have enabled us to receive invaluable feedback on improving clinical skills associated with sensitive scenarios. We do appreciate that due to a lack of resources, these workshops can be difficult to organize. Another useful resource recommended by our universities are palliative care guidelines on breaking bad news. Furthermore, in final year, students will attend a compulsory breaking bad news workshop. The workshop familiarizes students with the SPIKES model which consists of the following stages; set up, perception, invitation, knowledge, empathy, strategy, and summary.^2^ Final year students felt more confident about approaching sensitive discussions after this workshop. Consolidation of this method is tested in final year Objective Structured Clinical Examinations, so that students are prepared to approach difficult situations when they start working as junior doctors.

We understand that, as medical students, the opportunity to witness a patient’s final moments is rare. However, to better prepare, we were shown a video of a patient nearing the end of life in advance. The video emphasized clinical signs that we could use to anticipate future such occurrences.

Palliative care teaching is an essential component of the medical student’s curriculum. We believe that although it may not be possible to witness a death, there are alternative methods that can prepare you for coping with death and providing adequate palliation as a junior doctor.
